# Fatal sclerosing mesenteritis: a 7-year-old male autopsy case report

**DOI:** 10.4322/acr.2023.434

**Published:** 2023-05-31

**Authors:** Juan Carlos Celis Pinto, Lucía Hernández Peláez, Guillermo Mendoza Pacas, Juan Mayordomo Colunga, Milagros Balbín, Ana Pitiot, Héctor-Enrique Torres-Rivas, Verónica Blanco Lorenzo

**Affiliations:** 1 Hospital Universitario Central de Asturias (HUCA), Pathology Department, Oviedo, Asturias, Spain; 2 Hospital Universitario Central de Asturias (HUCA), Pediatric Department, Oviedo, Asturias, Spain; 3 Hospital Universitario Central de Asturias (HUCA), Pediatric Intesive Care Unit, Oviedo, Asturias, Spain; 4 Instituto de Investigación Sanitaria del Principado de Asturias, Oviedo, Asturias, Spain; 5 Centro de Investigación Biomédica en Red de Enfermedades Respiratorias, Barcelona, Cataluña, Spain; 6 Hospital Universitario Central de Asturias (HUCA), Medicine Laboratory, Oviedo, Asturias, Spain; 7 Instituto Universitario de Oncología del Principado de Asturias, Molecular Oncology Laboratory, Oviedo, Asturias, Spain; 8 Instituto de Investigación Biosanitaria de Asturias, Oviedo, Asturias, Spain

**Keywords:** Panniculitis, Peritoneal, Autopsy, Fatal Outcome, Pediatrics, Case Reports

## Abstract

Sclerosing Mesenteritis (SM) is a rare diagnosis, particularly in pediatric patients, and is typically non-fatal when appropriately treated. Although molecular and immunohistochemical alterations have been described, no pathognomonic signature has been identified for this entity. This report presents a case of a seven-year-old boy who suffered sudden cardiorespiratory arrest. Upon autopsy, he was found to have multicentric SM on the upper mesentery, which led to bowel wall thinning and abdominal bleeding with bacterial translocation. We performed comprehensive morphological, immunohistochemical, and molecular analyses. SM is an atypical disorder with diverse clinical manifestations, including a rare but potentially fatal course. Early diagnosis is critical, given its potential severity. To our knowledge, this is the first case report of pediatric mortality linked to SM. Our findings emphasize the importance of increased awareness and early detection of SM in pediatric patients.

## INTRODUCTION

Sclerosing Mesenteritis (SM) is a rare and complex entity with a multifaceted pathogenesis characterized by variable degrees of fibrosis, chronic inflammation, and fat necrosis. The condition was first described by Jura in 1924,^1^ and the frequency of SM ranges from 0.16-3.4%, depending on the series. To date, no specific incidence of SM reported in pediatric age.^2,3^

The literature indicates that SM is more common in Caucasian males with a history of abdominal surgery, trauma, or autoimmune disease.^3,4^ The first systematic review, conducted by Sharma et al. in 2017,^4^ included 192 cases and found that SM’s male-to-female ratio was 2.3:1, and a mean age of 55 ± 19.2 years.^4^ The review also found that symptoms such as intractable abdominal pain, bloating, intestinal obstruction, and massive ascites can last more than a month. Elevated levels of C-reactive protein (CRP) and erythrocyte sedimentation rate (ESR), along with leukocytosis, were the most common laboratory findings.^5-8^

While SM is rarely fatal, this case report presents a unique and unprecedented scenario of a fatal evolution of SM in Pediatrics. The patient's clinical course and symptoms were consistent with previously reported cases, including bowel obstruction and ileus development. However, the patient's condition progressively deteriorated as medical attention was not pursued, leading to a fatal outcome.

The ambiguity surrounding SM arises from the overlap between variable degrees of fibrosis, chronic inflammation, and fat necrosis. There are two main classifications: clinical pathological and histological. In 1974, Kipfer et al.^5^ proposed three categories that have become a de facto standard in the absence of gross definitive criteria: Type I, diffuse mesenteric thickening; Type II, single discrete tumor; and Type III, multiple discrete tumors.^5^ In 1997, Emory et al.^6^ reviewed 84 cases and proposed histological standards, dividing SM into three categories: mesenteric lipodystrophy, mesenteric panniculitis, and sclerosing (retractile) mesenteritis.^6^

The etiopathogenesis of SM remains unclear, but proposed etiologies include infection, ischemia, autoimmunity, paraneoplastic process, and abdominal surgery or trauma. This case report presents an opportunity to deepen our understanding of this exceptional condition’s etiopathogenesis and clinical course, particularly in pediatric age.^2,3,7-9^

## CASE REPORT

We present the case of a 7-year-old boy who experienced a sudden cardiorespiratory arrest at home. The patient had a past medical history of ileocolic intussusception at 22 months, which was treated using a sonographic-guided hydrostatic reduction. The family reported several recurrent episodes of abdominal pain over the last few years, but no medical care was sought.

On the day of the incident, the patient suddenly complained of abdominal pain and vomited coffee-ground-like material, followed by loss of consciousness. Upon arrival at the emergency, pulseless electrical activity, and severe abdominal distention were depicted. Prompt advanced cardiopulmonary resuscitation (CPR) was started, but nonreactive mydriasis was observed.

Further assessment revealed a hemoglobin level of 6.7 g/dL (11.5-15.5 g/dL) and a lactate level of 21 mmol/L, indicating severe anemia and hypoperfusion. An abdominal radiography was performed, which showed pneumoperitoneum. Despite more than 30 minutes of CPR, the patient was pronounced dead.

Given the unexpected nature of this event, an autopsy examination was solicited by the Pathology Department.

### Autopsy findings

During the corpse’s external examination, multiple bruises of approximately 2 cm in different stages of resolution were observed in the abdomen and thorax, accompanied by lividities. The abdomen was distended and firm by gas or fluid.

**Figure 1:**
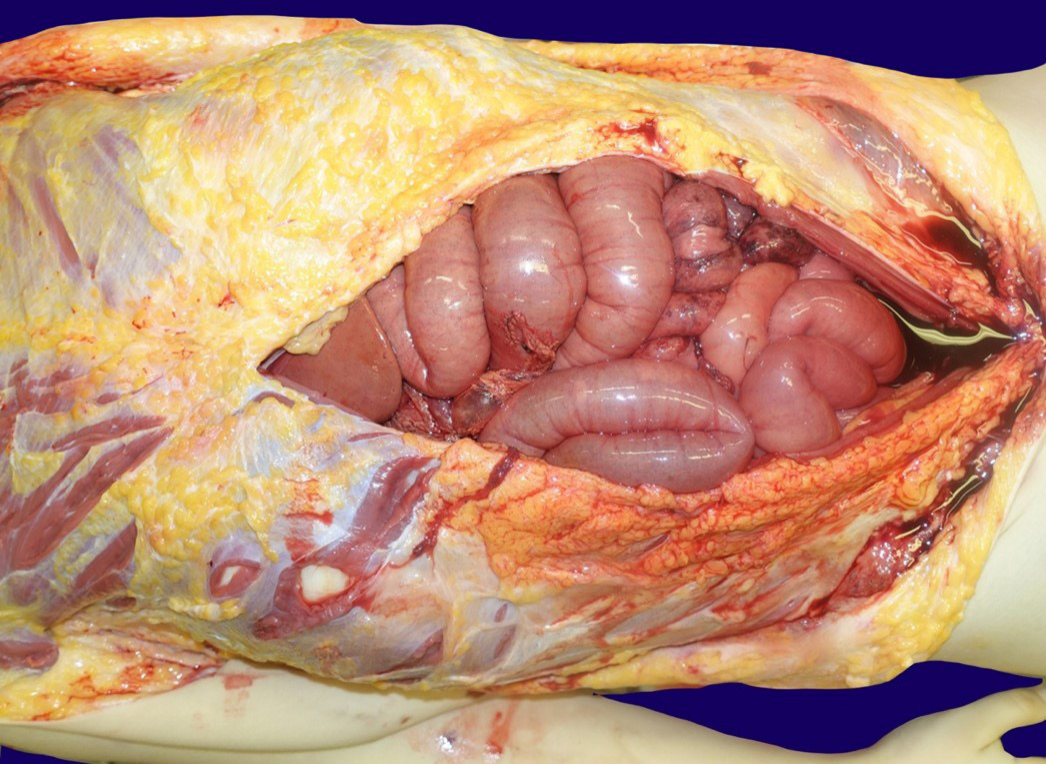
Gross view of the abdominal cavity overture depicts the exposed small bowel loops that are visibly filled with air due to CPR maneuvers. The presence of hemoperitoneum on the lower abdomen can be noted, filling the cavity. Additionally, we can observe the area of chronic damage on a non-perforated bowel loop with loss of serosa and exposure of inner layers. This image clearly depicts of the extent of abdominal pathology and highlights the damage sustained in both acute and chronic settings.

Upon exposing the abdominal cavity, a malodorous pressure gas was expelled, and approximately 2L of a bloody liquid was found surrounding the intestines. A small bowel loop was observed to be severely damaged without macroscopic perforation (Figure 1).

The gross examination of the intestinal loops revealed a retraction and induration of the mesenteric adipose tissue, measuring roughly 5x5cm, beneath the compromised small bowel loop. Furthermore, dozens of lymph nodes and discrete fibrous conglomerates of approximately 1-1.5cm were present in the upper mesentery.

Based on the findings, the preliminary cause of death was determined to be the 5x5cm mesenteric adipose tissue retraction area, which caused bowel wall thinning and subsequent abdominal bleeding.

### Gross and histopathological examination

**Figure 2:**
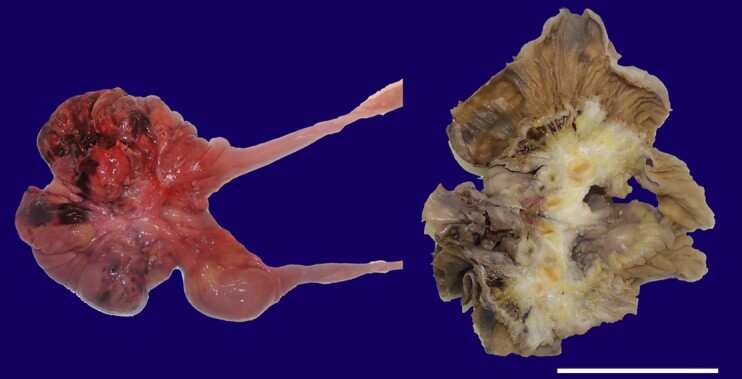
Gross view of the 20cm section of the affected bowel loop, with two images for comparison. On the left, a non-fixated picture clearly shows the retraction and bleeding. A formalin fixated picture after a mid-section cut is displayed on the right. Notably, the image on the right reveals the replacement of mesenteric adipose tissue with pearly-white tissue, engorging enlarged regional lymph nodes, the rectification of vasculature, and contact with the bowel wall. These enlarged regional lymph nodes have a yellowish appearance with a darker center. Scale bar= 10 cm.

The histopathological examination of the mesentery retraction and loop thinning was undertaken in a 20cm section fixated with a buffered-on 10% formalin solution (Figure 2).

After fixation, a mid-section cut was performed, revealing a somewhat fibrotic lesion. Representative sections were taken for microscopic evaluation, including the fibrous conglomerates and lymph nodes, which were resected and picked randomly.

**Figure 3:**
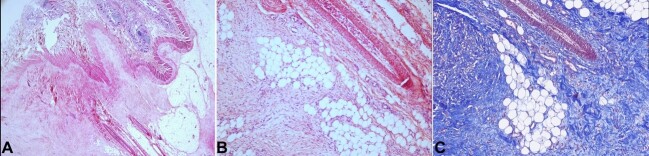
Microphotographs of the junction between the mesentery and bowel wall. **A**  **–** at low magnification power shows the lesion’s sclerosing nature with the fibroblast substitution of adipose tissue and rectification of vasculature. This lesion contacts the bowel wall in the upper-mid left field (H&E, 20X); **B**  **–** Lower image at a higher magnification power appreciating an admixture of fibroblasts engorging adipose tissue, with focal areas of fat necrosis and rectified vasculature (H&E, 100X); **C**  **–** Masson's trichrome staining in the same region as depicted in B. This image highlights the presence of variable-sized collagen bundles, which appear to be increased in number, interspersed, and engorging the surrounding adipose tissue (MT, 100X).

Following paraffin tissue embedding, 4-micron H&E-stained sections revealed a noteworthy proliferation of fibroblasts over a collagenous stroma, which surrounded and isolated isles of adipocytes, nerves, and vasculature, contacting the muscularis propria with associated extensive areas of hemorrhage. Focal areas of fat necrosis, inflammation, and hemosiderin deposition were also identified. The fibroblasts exhibited fine chromatin without evident nucleoli, pale eosinophilic cytoplasm, and no atypia or atypical mitosis (Figure 3).

**Figure 4:**
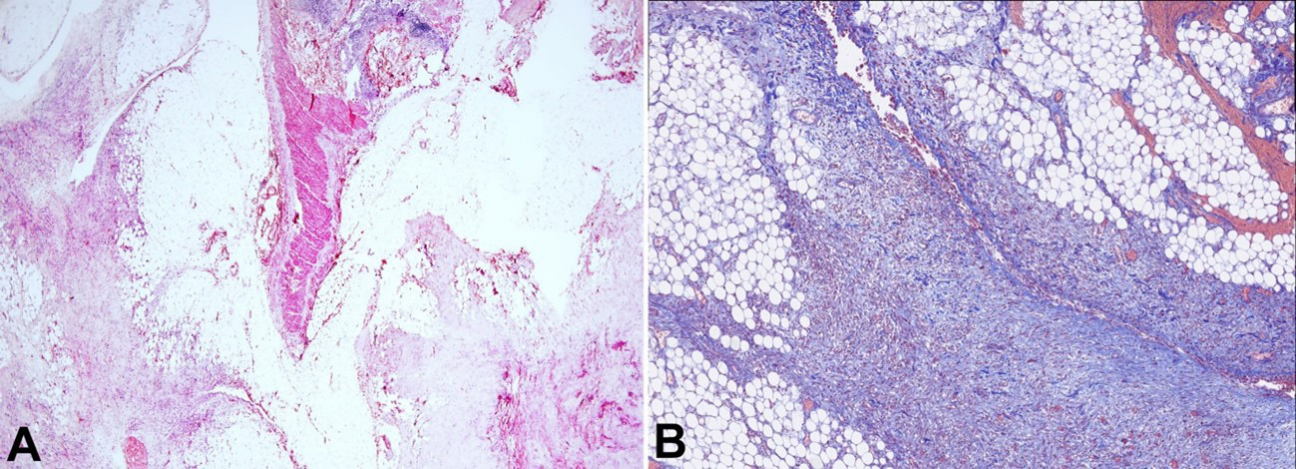
Microphotograph of a 1.5cm mesenteric distant mass that exhibits the same features as the primary lesion but on a smaller scale. Notably, the image showcases the admixture of fibroblasts with adipose tissue in the bottom left and bottom right fields. In the middle of the image, a tangential cut of the bowel wall is visible, with the mucosa positioned on top (H&E, 20X); **B**  **–** Higher magnification power at the bottom right field of **A**. Masson's trichrome staining emphasizing the heterogeneous distribution of collagen bundles, varying in size, which exhibit an increased density and are intermingled within the adipose tissue. Additionally, focal areas of hemorrhage can be discerned (MT 40X).

Similar findings were also observed, but at a smaller scale, along the small bowel mesentery (Figure 4).

**Figure 5:**
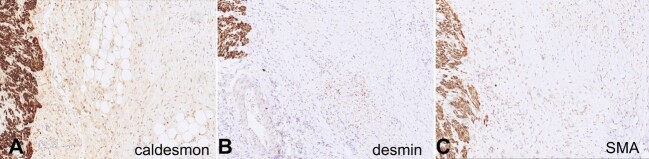
Microphotographs illustrating immunohistochemical staining for caldesmon (**A**), desmin (**B**), and smooth muscle actin (SMA) (**C**). The three images display a positive internal control on the left side, representing the muscularis propria of the bowel wall. In the center of each image, fibroblasts are observed, exhibiting either faint or focal staining positivity for these markers in comparison to the internal control. Multiple stains were employed to verify the positivity and intensity of these immunohistochemical stains (**A**, **B** and **C** **–** caldesmon, desmin, SMA 100X, respectively).

After a careful macro and micro-evaluation of the lesion, the differential diagnoses were pursued by ancillary studies. The differentials comprised desmoid tumor, familial adenomatous polyposis-associated desmoids, aggressive pediatric fibromatosis, lipofibromatosis, fibrous hamartoma of infancy, low-grade myofibroblastic sarcomas and fibrosarcomas. An immunohistochemistry panel was performed to test for desmin, smooth muscle actin (SMA), caldesmon, beta-catenin, CD-117, DOG-1, S100, Sox-10, CD34, STAT6, IgG4, p53, NTRK, and Ki67 (Figure 5).

Among these, only caldesmon, desmin, and SMA tested positive for fibroblast proliferation, and the proliferation index measured by Ki67 was between 1-2%. Molecular analysis was performed by next-generation sequencing to detect mutations in CTNNB1 (exon 3), BRAF codon 600, and hotspots mutation in 40 additional genes, using purified DNA from FFPE-tissue, with no mutations found. Multiplex ligation-dependent probe amplification (MLPA) was used for genes APC, MUTYH, and GREM1, and no copy number variations were detected. The Idylla GeneFusion Assay was used to determine expression imbalance in NTRK1, NTRK2, and NTRK3 genes, which can indicate gene fusion with no expression imbalance detection.

Based on the histological examination and the results of the various diagnostic tests, a diagnosis of SM was established.

## DISCUSSION

SM, or retractile mesenteritis, is a rare diagnosis and even rarer in Pediatrics. In our case, the patient was a Caucasian male, which aligns with previous studies, but the underlying cause of SM remains unknown. The primary theories about the etiology of SM center on abdominal trauma, particularly surgical trauma, and an IgG4-mediated autoimmune phenomenon. However, we believe that the ileocolic intussusception the patient experienced does not fit the abdominal trauma category, as hydrostatic reduction is a low-impact procedure that preserves normal anatomy. Full-body X-rays taken during the autopsy ruled out significant skeletal trauma or fractures. Additionally, the autoimmune theory was discarded due to the absence of a familial history of autoimmune diseases, and immunohistochemistry for IgG4 was negative.^8,9^

Establishing a diagnosis of SM is challenging because it is an exclusion diagnosis. Given the unfortunate circumstances in which we received our patient, the gross examination of the abdominal cavity, and histological appearance, a desmoid tumor was initially considered. However, this diagnosis was ruled out as no small bundles of infiltrative spindle cells in an abundant fibrous stroma were observed. IHC resulted in cytoplasmic for beta-catenin and negative for vimentin, respectively being only positive for SMA in the fibroblasts. As this IHC was not definitive, molecular analysis for CTNNB1 was obtained and found normal. Additional investigation into APC, MUTYH, and GREM1, copy number variations, was conducted, which excluded familial adenomatous polyposis (FAP) associated desmoids and aggressive pediatric fibromatosis.^10-14^

The lack of fibroblast infiltration beyond the mesentery, the absence of mitotic figures, nuclear atypia, and low Ki-67 directed the case towards a non-malignant entity. Several diagnoses were excluded, including gastrointestinal stromal tumors, solitary fibrous tumors, infantile fibrosarcomas, malignant peripheral nerve sheath tumors, lipofibromatosis, low-grade myofibroblastic sarcoma, fibrous hamartoma of infancy, and desmoid-type fibromatosis. Neither histology nor IHC results correlated with these entities as CD-117, DOG-1, STATE6, CD34, S100, SOX10, and NTRK resulted negative in the fibroblasts, and no fusions were observed in NTRK genes.^15,16^

SM's clinical presentation includes a wide range of non-specific symptoms, with abdominal pain being the most common and lasting up to 10 years before diagnosis.^8^ Imaging studies often reveal SM as an incidental diagnosis in CT scans obtained for other reasons, on which a “pseudo-capsule,” a “misty mesentery,” or a more specific “fat ring sign” may be observed. Medical treatments have mainly focused on tamoxifen and prednisone in patients who are not surgical candidates.^17^ In our case, the patient presented several years before death with moderate abdominal pain as a single symptom, and no CT scans were performed due to his age and mild symptoms. As a result, SM was not diagnosed in vivo, and no surgical or medical treatment was ever considered.

In addition to the primary lesion, other lesions with similar histological characteristics were observed in the upper mesentery. This multicentric presence of SM may align with a Type III Kipfer classification.^5^ While some studies have noted the association of lymphoma with SM, morphological analysis of the upper mesenteric lymph nodes was histologically normal.^9^ Our case represents an untreated pediatric Type III retractile SM that naturally evolved into a fatal outcome.^4,5^ The observed bowel alterations result from mesenteric fibrosis. This condition progressively obstructs blood flow to the farthest sections of the antimesenteric region. This obstruction compromises the tissue repair mechanisms, thereby creating a vicious cycle that eventually leads to erosion of the superficial layers, rupture of small blood vessels, low-volume hemorrhage, and translocation of bacteria into the peritoneum without any visible perforation.

Our assertions are supported by an array of evidence, encompassing the patient's clinical symptoms, identification of pneumoperitoneum through radiological imaging, and malodorous pressurized gas released during the opening of the abdominal cavity at autopsy. Interestingly, these observations were made despite the absence of any discernible perforations in the gastrointestinal tract.

We can only hypothesize about the development of pneumoperitoneum in this case. It is widely accepted that this condition results from a multifactorial process, likely manifesting in the final stages of a multi-year disease. Intense bowel thinning might permit direct gas diffusion into the peritoneal cavity, and the gas diffusion rate could have increased as CPR maneuvers intensified the internal pressure of the small intestines, as illustrated in Figure 1. Another feasible scenario could involve partial gas production by translocated bacteria, potentially accounting for the foul-smelling gas released during the autopsy. However, we currently lack any definitive evidence to support these theories, apart from the observation that malodorous gas had accumulated in the absence of visible macroscopic perforations, and there was no indication of fibrin deposition or histological signs of peritonitis.

The most affected areas of the bowel wall exhibited persistent low-volume bleeding, which ultimately accumulated to 2 liters by the time of death. This significant blood loss contributed to a hypovolemic and hypoperfused systemic state. The compromised circulation eventually led to the patient's collapse, characterized by abdominal pain, coffee-ground vomit, and loss of consciousness.

Additionally, acute and chronic damage has been documented and is visible in Figure 1, and the microscopic findings depicted in Figures 3 and 4. It's crucial to note that this chronic process is entirely treatable. However, if left unattended and allowed to develop fully, it could result in significant abdominal bleeding, hypovolemia, bacterial translocation, and ultimately, death. Therefore, swift and effective medical intervention is imperative.

Sclerosing mesenteritis is a rare condition with an unclear natural history due to a lack of understanding, inconsistent terminology in the literature, and inadequate follow-up. While cases of spontaneous regression have been reported, the resolution time varies widely. Advances in immunohistochemical and molecular analyses provide an opportunity to gain more insights into the etiology of this condition and its natural history. This case offers a unique opportunity to explore further and understand the natural history of sclerosing mesenteritis, which could lead to better diagnostic and treatment options.^7,18^

In recent years, there have been cases of SM in pediatric patients, including an infant who had SM but whose cause of death was not causally related to SM, and a boy in early childhood who underwent satisfactory treatment with Prednisolone.^19,20^ However, to the best of our knowledge, this report represents the first case of a death that is causally related to SM in a pediatric patient.
